# The pivotal role of ECG in cardiomyopathies

**DOI:** 10.3389/fcvm.2023.1178163

**Published:** 2023-06-19

**Authors:** Elisa Silvetti, Oreste Lanza, Fabiana Romeo, Annamaria Martino, Elisa Fedele, Chiara Lanzillo, Cinzia Crescenzi, Francesca Fanisio, Leonardo Calò

**Affiliations:** Division of Cardiology, Policlinico Casilino, Rome, Italy

**Keywords:** electrocardiogram (ECG), dilated cardiomyopathy, arrhythmogenic cardiomyopathy, hypertrophic cardiomyopathy, risk stratification, diagnosis

## Abstract

Cardiomyopathies are a heterogeneous group of pathologies characterized by structural and functional alterations of the heart. Recent technological advances in cardiovascular imaging offer an opportunity for deep phenotypic and etiological definition. Electrocardiogram (ECG) is the first-line diagnostic tool in the evaluation of both asymptomatic and symptomatic individuals. Some electrocardiographic signs are pathognomonic or fall within validated diagnostic criteria of individual cardiomyopathy such as the inverted T waves in right precordial leads (V1–V3) or beyond in individuals with complete pubertal development in the absence of complete right bundle branch block for the diagnosis of arrhythmogenic cardiomyopathy of the right ventricle (ARVC) or the presence of low voltages typically seen in more than 60% of patients with amyloidosis. Most other electrocardiographic findings such as the presence of depolarization changes including QRS fragmentation, the presence of epsilon wave, the presence of reduced or increased voltages as well as alterations in the repolarization phase including the negative T waves in the lateral leads, or the profound inversion of the T waves or downsloping of the ST tract are more non-specific signs which can however raise the clinical suspicion of cardiomyopathy in order to initiate a diagnostic procedure especially using imaging techniques for diagnostic confirmation. Such electrocardiographic alterations not only have a counterpart in imaging investigations such as evidence of late gadolinium enhancement on magnetic resonance imaging, but may also have an important prognostic value once a definite diagnosis has been made. In addition, the presence of electrical stimulus conduction disturbances or advanced atrioventricular blocks that can be seen especially in conditions such as cardiac amyloidosis or sarcoidosis, or the presence of left bundle branch block or posterior fascicular block in dilated or arrhythmogenic left ventricular cardiomyopathies are recognized as a possible expression of advanced pathology. Similarly, the presence of ventricular arrhythmias with typical patterns such as non-sustained or sustained ventricular tachycardia of LBBB morphology in ARVC or non-sustained or sustained ventricular tachycardia with an RBBB morphology (excluding the “fascicular pattern”) in arrhythmogenic left ventricle cardiomyopathy could have a significant impact on the course of each disease. It is therefore clear that a learned and careful interpretation of ECG features can raise suspicion of the presence of a cardiomyopathy, identify diagnostic “red flags” useful for orienting the diagnosis toward specific forms, and provide useful tools for risk stratification. The purpose of this review is to emphasize the important role of the ECG in the diagnostic workup, describing the main ECG findings of different cardiomyopathies.

## 1. Introduction

Cardiomyopathies are defined as a heterogeneous group of pathologies characterized by structural and functional alterations of the heart in the absence of coronary artery disease (CAD), hypertension, valvular disease, and congenital heart disease sufficient to explain the observed myocardial abnormality. They are grouped into specific morphological and functional phenotypes, with each phenotype subclassified into familiar/genetic and non-familiar/non-genetic forms. Non-genetic cardiomyopathies include idiopathic forms with no identifiable cause and acquired forms associated with systemic disorders (1).

**Table 1:** Main ECG findings in cardiomyopathies.

	DCM/HDCM	NMD	ACM	HCM	AFD and storage disease	CA	Sarcoidosis
Depolarization abnormalities	fQRS; pathological Q-wave; occasionally LQRSV (e.g., in FLNC mutation)	LQRSV in the presence of scar	fQRS; epsilon wave; SAECG; TAD > 55 ms in V1; LQRSV in limb leads	Sokolow–Lyon (V1 S onda + V5 o V6 R dell'onda ≥ 35 mm); Cornell signs (SV3 + RaVL with a cutoff for LHV >2.0 mV in women and >2.8 mV in men); LQRSV (if extensive LGE); Q waves; fQRS	Q waves; fQRS; extremely high voltages (Danon disease)	LQRSV; QS pattern in precordial leads	fQRS; epsilon wave
AV conduction disorders	First-degree and advanced AVB especially in some defined genetic form (e.g. involving LMNA gene)	Advanced AVB	—	—	Short PR interval (AFD; Danon disease)	First-degree and advanced AVB	Advanced AVB
IV conduction disorders	RBBB (rare); LBBB	LBBB	RBBB-like pattern (ARVC); LPFB (ALVC)	—	—	IV conduction delay	RBBB
Repolarization abnormalities	TWI in inferior, antero-lateral, and infero-lateral leads	TWI in infero-lateral leads	TWI in right precordial (ARVC); TWI in lateral leads	ST strain (≥1 mm concave downsloping ST-segment depression); asymmetrical TWI in lateral leads; “pseudo-STEMI” pattern (ST-segment elevations in antero-lateral leads); deep T-wave inversion (≥2 mm) in infero-lateral leads; “giant T waves” (>10 mm), in the apical form of HCM	ST-segment depression and TWI in infero-lateral leads	ST-segment depression and TWI in infero-lateral leads	TWA; higher T-wave amplitude in lead aVR; TWI, and a longer interval of T-peak to T-end; QT dispersion
P-wave morphology alterations	Increased P-Wave duration and dispersion	—	—	Bifid and large P-wave (total duration > 110 ms); P-wave amplitude > 2.5 mm in inferior leads and > 1.5 mm in V1–V2	—	—	—
Arrhythmias	AF; VT	AF; VT; BBR-VT	VT with LBBB pattern (ARVC); VT with RBBB pattern (ALVC)	AF; VT often with RBBB morphology	AF; VT	AF	VT

AF, atrial fibrillation; AFD, Anderson–Fabry disease; ACM, arrhythmogenic cardiomyopathy; ALVC, arrhythmogenic left cardiomyopathy; ARVC, arrhythmogenic right ventricle cardiomyopathy; AVB, atrioventricular block; BBR-VT, bundle branch reentry ventricular tachycardia; CA, cardiac amyloidosis; DCM/HDCM, dilated cardiomyopathy/hypokinetic dilated cardiomyopathy; FLNC, filamin C; fQRS, fragmented QRS; IV, intraventricular; LBBB, left bundle branch block; LMNA, lamin A; LPFB, left posterior fascicular block; LQRSV, low QRS voltage; NMD, neuromuscular dilated; RBBB, right bundle branch block; TAD, terminal activation delay; TWA, T-wave alternans; TWI, T-wave inversion; VT, ventricular tachycardia.

While much progress has been made in recent years in early diagnosis and treatment, cardiomyopathies still remain the diseases with high mortality and morbidity (2). In 2013, the ESC Working Group on Myocardial and Pericardial Diseases proposed a diagnostic workup that can guide the clinical approach in cardiomyopathies, based on the recognition of diagnostic “red flags” (3).

The approach to the diagnosis of cardiomyopathies includes not only sophisticated tests such as genetic testing and imaging tests but also electrocardiogram (ECG). This latter provides important information for orienting the diagnosis toward specific forms. The electrocardiogram is used as a first-line diagnostic tool in the evaluation of both asymptomatic and symptomatic individuals. It is often the first test to suggest the possibility of a myocardial disease, and its analytical interpretation, associated with the assessment of clinical context, may enable the early identification of specific genetic or acquired forms of cardiomyopathies. In addition, the identification of specific ECG abnormalities can provide information about the severity of the disease and represent a useful diagnostic tool for appropriately directing subsequent clinical and therapeutic decisions (4).

In this review, we have summarized the main ECG abnormalities that can be found in different cardiomyopathies, focusing on cardiomyopathy-specific “red flags” (Table 1).

## 2. Dilated cardiomyopathy

Dilated cardiomyopathy (DCM) is defined by the presence of left ventricular (LV) or biventricular dilatation and systolic dysfunction in the absence of abnormal loading conditions (hypertension, valve disease) or coronary artery disease sufficient to cause global systolic impairment (1). This pathology includes a broad range of genetic and acquired disorders manifesting as a spectrum of electrical and functional abnormalities that change with time. Therefore, it has recently been proposed to update the criteria for diagnosis of DCM, including intermediate phenotypes, “hypokinetic non-dilated cardiomyopathy,” that do not meet the standard definition of DCM (5).

To better investigate the great phenotypic heterogeneity and the different etiologies, the diagnostic workup of DCM includes a history and physical examination, laboratory tests, electrocardiogram, cardiac imaging, and genetic tests (3).

Several studies have described the ECG findings in patients with DCM, showing that the ECG is rarely normal in these patients and the presence of ECG abnormalities should trigger the initiation of a diagnostic pathway (3–6). The most common ECG findings include left ventricular hypertrophy (LVH), T-wave inversions (TWI), left axis deviation, pathological Q waves, and conduction alterations. While such ECG abnormalities were long considered non-specific for DCM patients, newly acquired knowledge on genotype–phenotype correlations has now made the ECG a useful tool to guide the etiological diagnosis (“red flags”) and provide prognostic stratification.

### 2.1. ECG abnormalities and arrhythmias

Studies on the correlation between electrocardiographic and echocardiographic findings have well demonstrated that P-wave abnormalities are an expression of atrial enlargement due to increased filling pressures and are associated with valvular abnormalities (7, 8). Maximum P-wave duration and P-wave dispersion (PWD), defined as the difference between maximum and minimum P-wave duration, have been found to be higher in patients with dilated cardiomyopathy than in healthy control subjects (9). These anatomical and structural abnormalities underlie the increased risk of developing atrial fibrillation (AF) in patients with DCM (10).

Several depolarization and repolarization abnormalities have been reported in patients with DCM (11, 12). In literature, the first-degree atrioventricular (AV) block has a prevalence of 10%–23% in the DCM population, although advanced AV blocks can also be found in these patients (12). The involvement of the AV node or His–Purkinje system, especially in the young population, should raise the suspicion of specific genetic diseases involving the lamin A/C gene, SCN5A gene, and variants in the emerin gene. In these patients, conduction disturbance may precede LV dysfunction and play an important role in risk stratification for sudden death (13). Furthermore, conduction anomalies are frequent in acquired conditions such as myocarditis and sarcoidosis (3).

**Figure 1:**
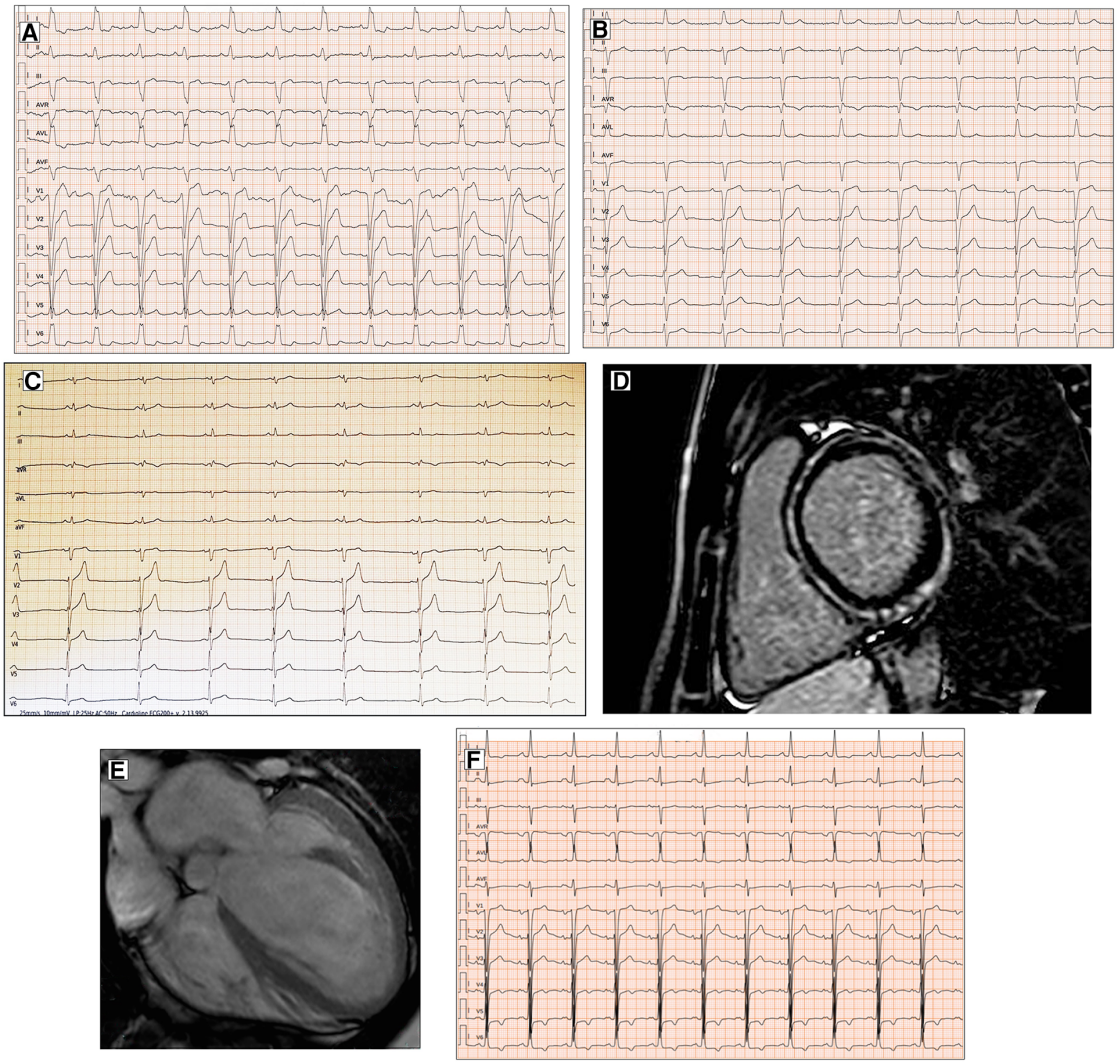
Conduction disorders in patients with DCM. ECG performed in a 60-year-old man with DCM shows LBBB and normal QRS axis (**A**). Basal ECG of a 78-year-old patient fulfills criteria for LAFB (**B**). ECG and CMR findings of a 20-year-old male with history of ventricular arrhythmias and mild LV dysfunction. Basal ECG (**C**) showing low QRS voltages (<0.5 mV) in limb leads and LPFB (AQRS ≈ + 110°). Post-contrast image showing subepicardial circumferential LGE pattern (**D**). Four-chamber CMR image of a 42-year-old man presented with pulmonary edema showing severe LV dilation (**E**). ECG at presentation displaying normal QRS axis, intraventricular conduction delay, signs of LVH (Sokolow–Lyon criteria) with secondary repolarization abnormalities (**F**). All the ECGs presented in the figure were performed at 25 mm/s with 1 mm/mV. ECG, electrocardiogram; CMR, cardiac magnetic resonance; DCM, dilated cardiomyopathy; LAFB, left anterior fascicular block; LBBB, left bundle branch block; LGE, late gadolinium enhancement; LPFB, left posterior fascicular block; LVH, left ventricular hypertrophy.

Right bundle branch block (RBBB) has a prevalence of 2%–6% among patients with DCM and is frequently associated with neuromuscular disorders (12). Left bundle branch block (LBBB) is far more common than RBBB. LBBB is present in approximately one-third of DCM patients (14) (Figure 1A). Defined by Strauss et al. (15) as a QRS duration of ≥140 ms in men and ≥130 ms in women; QRS notching or slurring in two or more contiguous leads of V1–V6, I, and aVL; and a QS or rS in V, LBBB may appear in the natural course of DCM, representing a marker of disease severity, although it sometimes occurs at the onset preceding the structural phenotype. Several studies have previously demonstrated the negative prognostic impact of LBBB, with an increased risk of all-cause mortality, by determining an asynchronous contraction of the LV and a progressive worsening of systolic function (16). LBBB is the target of cardiac resynchronization therapy (CRT) that has been shown to be able to improve cardiac function, symptoms, and well-being and to reduce morbidity and mortality in an appropriately selected group of DCM patients (17).

In addition, left anterior fascicular block and non-specific intraventricular conduction delay, while not being specific ECG signs, have also been found in a small percentage of DCM patients (12) (Figure 1B). Left posterior fascicular block (LPFB), which is uncommon in the general population, has been associated in a recent small study with extensive LV scarring and an increased risk of sudden death (18) (Figure 1C,D).

Regarding LVH, the literature describes a presence ranging from 17% to 69% according to the Sokolow criteria (12) (Figure 1E,F). In DCM patients with LVH voltage criteria (6), a hypertensive etiology should be excluded. Interestingly, in a retrospective study by Merlo et al. (14), LVH showed a protective role, probably expressing a prognostic benefit due to an increased left ventricular mass.

In 1982, Goldberger (19) described an ECG triad (“Goldberger’s triad”) consisting of (1) SV1 or SV2 + RV5 or RV6 of > 3.5 mV, (2) total QRS amplitude in each of the limb leads of 0.8 mV, and (3) R/S ratio of <1 in lead V4, which had a positive predictive value of >90% and specificity of >90% for detecting severe (LVEF < 35%) LV systolic dysfunction. Later, Lopez et al. (20) suggested the utility of Goldberger's triad to identify patients with idiopathic DCM from hypertensive cardiomyopathy. The diagnostic role of this triad is currently well established only in patients with severe LV dysfunction, but it is unknown for patients with mild dysfunction or in the preclinical/asymptomatic stage.

**Figure 2:**
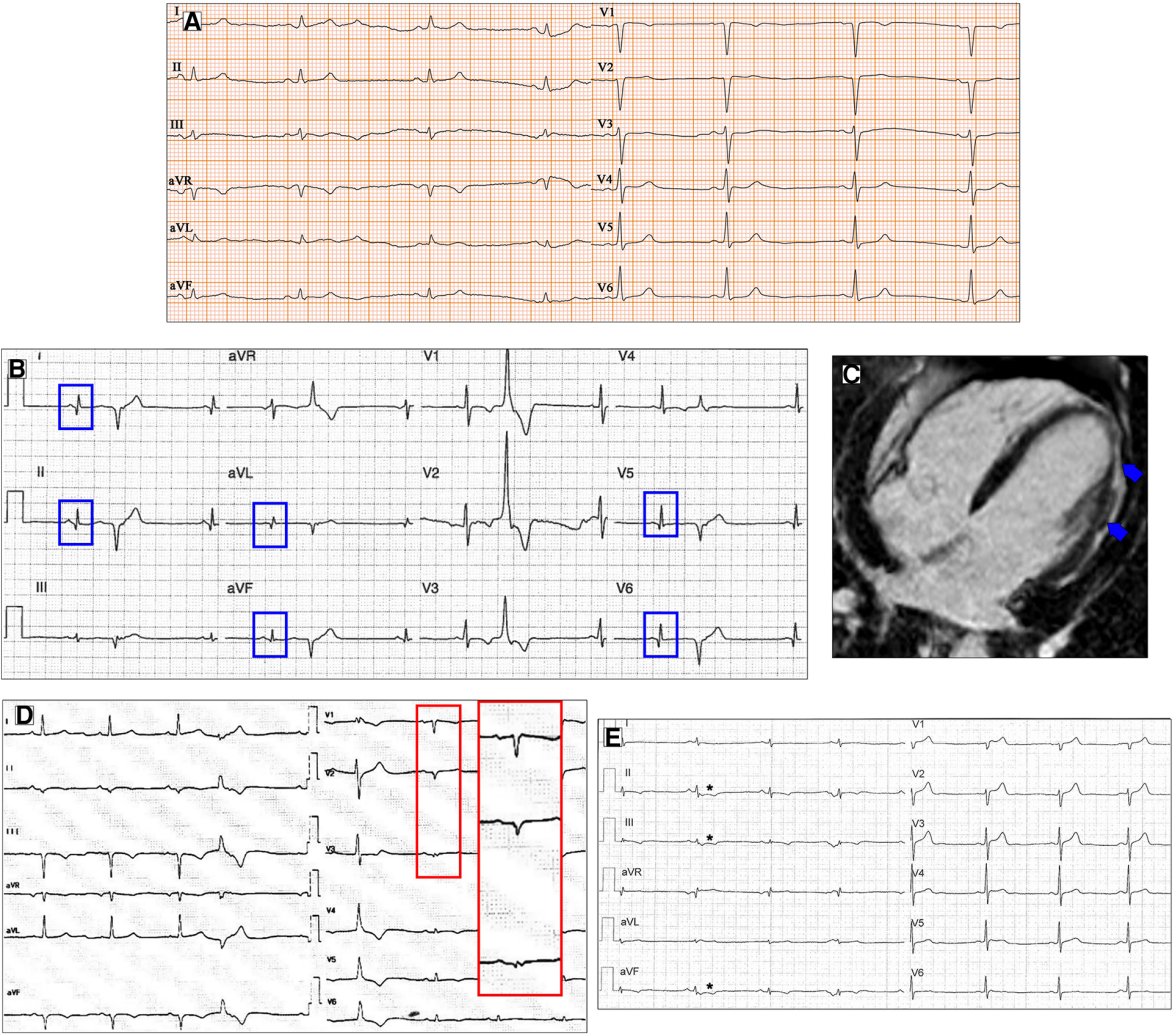
Ventricular depolarization and repolarization abnormalities in patients with DCM. A 30-year-old female with a likely pathogenic variant in FLNC gene. Twelve-lead ECG shows low QRS voltages both in limb and precordial leads (**A**). Basal ECG of a 46-year-old patient with DCM showing pathological infero-lateral Q waves (**blue boxes**, **B**); a premature ventricular beat with RBBB morphology and superior axis is also present. Post-contrast CMR images of patient in panel B showing subepicardial LGE involving the left ventricular lateral wall (**blue arrows**, **C**). A 38-year-old male with a pathogenic variant in LMNA gene and familiar history of DCM and sudden cardiac death. Basal ECG displaying low QRS voltages in precordial leads and specific ECG signs of “septal remodeling” (pathological Q waves, QRS fragmentation, poor R-wave progression in V1–V3 leads; **red box**); a premature ventricular beat with RBBB morphology and right axis deviation is also present (**D**). Basal ECG of a 39-year-old DSP mutation carrier shows low QRS voltages in limb leads, negative T waves in inferior leads (**asterisks**, **E**). All the ECGs presented in the figure were performed at 25 mm/s with 1 mm/mV. ECG, electrocardiogram; CMR, cardiac magnetic resonance; DCM, dilated cardiomyopathy; LGE, late gadolinium enhancement; RBBB, right bundle branch block.

Low electrocardiographic QRS voltages (LQRSV) are defined in the literature as a nadir-to-peak QRS amplitude of <5 mm in all limb leads and <10 mm in all precordial leads (21). In DCM patients, LQRSV have been described in 6% and may be observed only in limb leads (most frequently, Figure 1C), in precordial leads, or both (14). The prevalence of LQRSV increases in specific genetic etiologies. About 25% of carriers show low voltages in limb leads in filamin C (FLNC) mutations (Figure 2A), while in PLN mutations, low QRS complexes can be found in 46% of carriers, mainly in anterior–lateral precordial leads (22, 23). These ECG anomalies are considered “red flags” that should suggest the diagnosis of these particularly aggressive genetic forms with a high risk of arrhythmias and sudden cardiac death (SCD). In the absence of other possible causes, LQRSV likely reflect the loss of vital myocardium and its replacement by the fibrotic tissue, reducing QRS amplitude, especially in precordial leads (14, 24). This has been well demonstrated by Oloriz et al. (25), who found a relationship between r in V3 of ≤0.3 mV and QRS of <0.6 mV in inferior leads and the presence of fibrosis in the LV antero-septal and infero-lateral region on the endo-epicardial voltage maps. Furthermore, Rijdt et al. (26) demonstrated the association between low voltages and inverted lateral T waves at the ECG and the presence of LV late gadolinium enhancement (LGE) on MRI in PLN p.Arg14del mutation carriers.

Besides their diagnostic value, LQRSV play a prognostic role in clinical practice, as observed by Merlo et al. (14), who proposed a lower amplitude of R-wave in lead II + S wave in V2 + inverted T waves in antero-lateral leads as a new prognostic tool and an independent predictor of malignant ventricular arrhythmias (VA) and sudden cardiac death.

Several criteria are used to diagnose the presence of Q waves: Q-wave duration of ≥40 ms, absolute depth of >3 mm, or amplitude of ≥25% of the ensuing R-wave (27).

Q waves have been described more frequently in anterior and lateral leads in DCM, despite normal coronary arteries (14) (Figure 2B,C). ECG signs of “septal remodeling,” such as pathological Q waves in leads V1–V2, have been described in Lamin A/C (LMNA) mutation carriers (28) (Figure 2D). The presence of a pseudoinfarction pattern in posterior, postero-lateral, and inferior leads should suggest dystrophin-related disease (29).

Fragmented QRS (fQRS) is a marker of depolarization abnormality present in a significant number of patients with DCM (more than 20%) (14). Das et al. (30) defined fQRS as narrow QRS complexes with the presence of an additional R-wave (R′) or notching in the nadir of the R-wave or the S wave or the presence of >1 R′ (fragmentation) in two contiguous leads. In a wide QRS complex, fQRS is defined as the QRS complex with >2 R′ waves or notches in the R or S wave in two contiguous leads (BBB, paced QRS, or premature ventricular complexes).

The fQRS is probably the expression of disorganized and slowed conduction across the region of myocardial fibrosis. Some studies have shown a concordance between fQRS segment and the presence of LGE in the same localization at MRI (24, 31). In contrast, Ahn et al. (32) reported that fQRS was not correlated with LGE despite finding a significant poor prognosis in patients with DCM and fQRS. The prognostic role of fQRS in patients with CAD has been well established while remaining disputed in DCM patients (33). The study of Sha et al. (34) also confirmed the high predictive value for the combined endpoint of all-cause mortality and ventricular tachyarrhythmias of fQRS in DCM patients with left ventricular dysfunction. Conversely, in the prospective investigation of Cheema et al. (35), the presence of fQRS on ECG was not associated with a higher risk of either all-cause or arrhythmic mortality. The fQRS has also been shown to be associated with significant intraventricular dyssynchrony in patients with non-ischemic cardiomyopathy, narrow QRS, and sinus rhythm (36); such findings suggest the possibility of using fQRS as a predictor in identifying patients who can benefit from CRT, but these data have not yet been confirmed.

Repolarization abnormalities are very common in DCM patients and are an expression of the heart muscle involvement in the disease process. TWI was described as a T inversion of ≥0.1 mV in depth in ≥2 contiguous leads, in the absence of LBBB (21). The prevalence of TWI in the DCM population, as reported by the literature, is 15%–45% (37). The leads most frequently presenting TWI are inferior, antero-lateral, and infero-lateral. TWI in infero-lateral leads is a common finding in patients with FLNC and desmosomal variants (22) (Figure 2E). Merlo et al. (14) found in their study that on multivariate analysis, the presence of TWI in antero-lateral leads was an independent risk factor for sudden cardiac death and malignant ventricular arrhythmias; this result probably expresses the existence of overlapping phenotypes between DCM and arrhythmogenic ventricular cardiomyopathy with high mortality risk. The QT interval, while generally normal in DCM patients, has been shown to be of potential use in sudden cardiac death risk stratification in DCM when abnormal (38).

Patients with DCM may develop supraventricular and ventricular arrhythmias. AF is the most common supraventricular arrhythmia in this population, with a prevalence of 2%–40% (12). Its occurrence during follow-up is an unfavorable prognostic marker, associated with increased morbidity and mortality, probably due to structural disease progression (39). LMNA mutation carriers show a high prevalence of atrial arrhythmia, with AF present in almost half of these patients at their first presentation, often preceding the development of the dilated phenotype (40). Early-onset DCM and AF are also associated with SCN5A mutations, with a high prevalence (41). A study by Tayal et al. (42) on 572 prospectively recruited DCM patients found that only truncating variants in titin (TTNtv) predicted arrhythmic DCM [AF, ventricular tachycardia (VT), or non-sustained VT].

Any variety of VA can be found in DCM patients, from PVCs and non-sustained and sustained monomorphic ventricular tachycardia (NSVT and SVT, respectively) to polymorphic ventricular tachycardia and ventricular fibrillation (VF). PVCs and NSVT may be found in up to 40% of patients with DCM, but their role is not clear in the literature (12). It is well established that the frequency of arrhythmias increases with the severity of heart failure, worsening of the ejection fraction, and the New York Heart Association (NYHA) class (43, 44). Recent data suggest that both genetic and MRI findings can contribute to risk stratification (45).

In fact, it has been observed that the arrhythmogenic substrate is represented by areas of replacement fibrosis, which can be well-assessed by MRI with the identification of regions of LGE in the mid-wall or subepicardial region, favoring the origin and maintenance of VA. A meta-analysis of 29 studies, combining 2,948 patients with DCM, observed that LGE was significantly associated with the arrhythmic endpoint. Interestingly, the association between LGE and the arrhythmic endpoint remained significant among patients with mean LVEF of ≥35% (46).

Another promising tool for risk stratification is genetic analysis, as some genetic defects, such as LMNA, FLNC, RBM20, and PLN mutations, are associated with both LV dysfunction and polymorphic ventricular arrhythmias, with an increased risk of SCD. When these genetic defects are associated with other risk factors, such as mild or intermediate LV dysfunction, syncope, LGE on MRI, and inducible SMVT at PES, they represent a IIa C recommendation for implantable cardioverter defibrillator (ICD) implant, according to the European Guidelines (45). It is important to emphasize that genetic analysis is indicated in patients with DCM with atrioventricular conduction disorders at an age of less than 50 years or if there is already a family history of DCM or sudden cardiac death at an early age (also less than 50 years) (45–47). Furthermore, it is very relevant to specify how the presence of an LMNA mutation has a significant prognostic impact and therefore also determines a different management of therapeutic indications (48). In fact, in the presence of such a mutation, the indication for defibrillator implantation in addition to the classic indications for all other forms of DCM is also extended to patients with a risk of ventricular arrhythmias according to a dedicated score of >10% and with global systolic function of <50% or atrioventricular conduction disturbances or in the presence of NSVT (49).

### 2.2. ECG in neuromuscular diseases

Neuromuscular diseases are a heterogeneous group of disorders that affect both the neuromuscular system and the heart, often leading to DCM. The most common neuromuscular diseases involving the heart are myotonic dystrophy type 1 and 2, Duchenne muscular dystrophy (DMD), and Emery–Dreifuss muscular dystrophy type 1 and 2.

Myotonic dystrophy (MD) is a muscular dystrophy characterized by progressive muscle loss and weakness, caused by a genetic mutation of the Dystrophy myotonic protein kinase (DMPK) or the CCHC-type zinc finger nucleic acid binding protein (CNBP) genes (MD1 and MD2, respectively). Cardiac involvement in MD mostly affects the conduction system but may also include supraventricular and ventricular arrhythmias as well as LV systolic dysfunction and myocardial scar.

**Figure 3:**
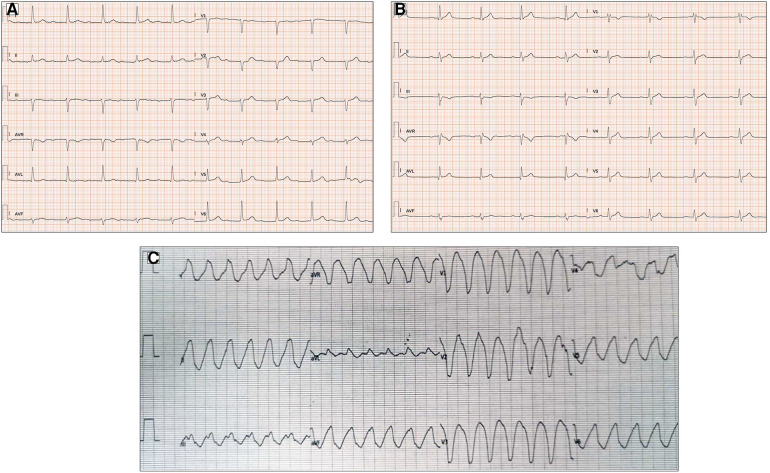
ECG abnormalities in patients with myotonic dystrophy type I. (**A**) Twelve-lead ECG in a 51-year-old patient shows prolongation of the PR interval (400 ms). (**B**) First-degree atrioventricular block, right bundle branch block, and pathological lateral Q waves in a 56-year-old patient. (**C**) Left bundle branch morphology regular wide tachycardia in a 52-year-old patient with history of arrhythmic storm. All the ECGs presented in the figure were performed at 25 mm/s with 1 mm/mV. ECG, electrocardiogram.

AV and interventricular (IV) blocks have been identified in up to 45% and 20% of MD1 patients, respectively (50) (Figure 3A,B). A PR interval of >240 ms and QRS duration of >120 ms on ECG, as well as second- or third-degree AV block, have been demonstrated to independently predict the risk of SCD in MD1 subjects (51). However, even milder AV or IV blocks (i.e., PR and QRS intervals of 200–240 ms and 100–120 ms, respectively) are considered “red flags” and, when associated with symptoms consistent with bradycardia, may lead to the performance of an invasive electrophysiologic study (EPS) for risk stratification and eventual pacemaker (PMK) or automatic implantable cardioverter defibrillator (AICD) implantation (47). Previous studies have shown that slightly prolonged PR intervals (>200 ms) are also independent predictors of an HV interval of >70 ms on EPS, a well-known marker of advanced His–Purkinje conduction system disease (52).

Unfortunately, AV and IV conduction intervals on ECG may be normal in up to 15.2% of MD1 patients with HV intervals of >70 ms on EPS, and, on the contrary, up to 66.1% of MD1 subjects with red flags on ECG may have normal HV intervals at EPS (53). Consequently, ECG should be combined with other clinical and structural parameters to identify more accurately MD subjects at risk of life-threatening bradyarrhythmias.

AF and atrial flutter may be present in up to 12% of MD subjects, even in the early stages of the disease, often preceding the diagnosis, and have been identified as predictors of SCD (48).

Finally, non-sustained and sustained VT may be present in up to 4.1% and 2.7% of MD1 and MD2 subjects, respectively (47) (Figure 3C). Pathophysiological mechanisms favoring VT include reentry circuits promoted by fibrotic foci, fatty infiltration, and delayed conduction in the His–Purkinje system. The occurrence of sustained and non-sustained VT may be predictive of subsequent malignant tachyarrhythmias in subjects with MD1, in contrast to the inducibility of VT during an EPS study, which has a limited value in the risk stratification of these subjects (54).

DMD is a recessive X-linked and autosomal recessive disorder characterized by symmetric myasthenia and amyotrophy. RBBB is common in children with DMD. Further significant abnormal ECG signs, including ST-segment changes, T-wave inversion, and mostly Q waves in the infero-lateral leads, which are the marker of intramyocardial scar, and prolonged IV conduction, may precede cardiomegaly and left ventricular dysfunction (55).

Sinus bradycardia is rare, but it may develop over time, as well as atrial arrhythmias. Advanced second-degree and most forms of third-degree AV block are associated with an adverse prognosis, even in asymptomatic individuals.

Emery–Dreifuss muscular dystrophy type 1 (EDMD1) and type 2 (EDMD2) are caused by mutations in the STA gene (encoding the nuclear membrane protein emerin) and in the LMNA gene, respectively. The ECG of EDMD1 patients is often characterized by sinus bradycardia, low-amplitude P waves, and a prolonged PR interval. Later on, AF, atrial flutter, complete AV block, and VT may develop, and they are associated with a worsening of prognosis (47).

## 3. Arrhythmogenic cardiomyopathy

The original definition of arrhythmogenic right ventricular dysplasia (ARVD), subsequently defined as arrhythmogenic right ventricular cardiomyopathy (ARVC), has long since been expanded and modified by subdividing the clinical and nosological entities of arrhythmogenic cardiomyopathies (ACM) according to the predominantly affected heart chamber (56–58). Today, arrhythmogenic cardiomyopathies are divided into a phenotype that predominantly or exclusively affects the right ventricle (ARVC), a phenotype that affects predominantly or exclusively the left ventricle (ALVC), and a phenotype with biventricular balanced involvement (59). The diagnostic criteria for defining the clinical phenotypes have also changed accordingly (55). The ECG remains one of the first and most effective instrumental approaches for the diagnostic suspicion of arrhythmogenic cardiomyopathy. As the definition has evolved and the field has been enriched with new nosological entities, the electrocardiographic criteria have also undergone a concomitant evolution with the possibility of distinguishing electrocardiographic features peculiar to arrhythmogenic cardiomyopathy of the right ventricle and the left ventricle, and when these same electrocardiographic features are associated, they may be the expression of a biventricular form (54).

### 3.1. ECG abnormalities and ventricular arrhythmias in ARVC

Various criteria, including electrocardiographic criteria, have been drawn up over the years to highlight this nosological development. In particular, with the new “Padua Criteria,” some of the electrocardiographic findings have been reclassified into the different forms of arrhythmogenic cardiomyopathy (54, 55). Independent of each electrocardiographic finding and its sensitivity and specificity value in the diagnosis of the different forms of ACM, the presence of these ECG findings may be the expression of an underlying substrate in which the ventricular wall is composed of vital myocardium and regions of fibro-fatty replacement of the myocardium itself (60). This can cause QRS complex fragmentation or prolongation and could predispose to the trigger of ventricular arrhythmias (61, 62). Loss of desmosomal protein could also be the cause of electrical instability due to the cross-talk between the structural proteins and the voltage-gated channels or the gap junctions (63).

**Figure 4:**
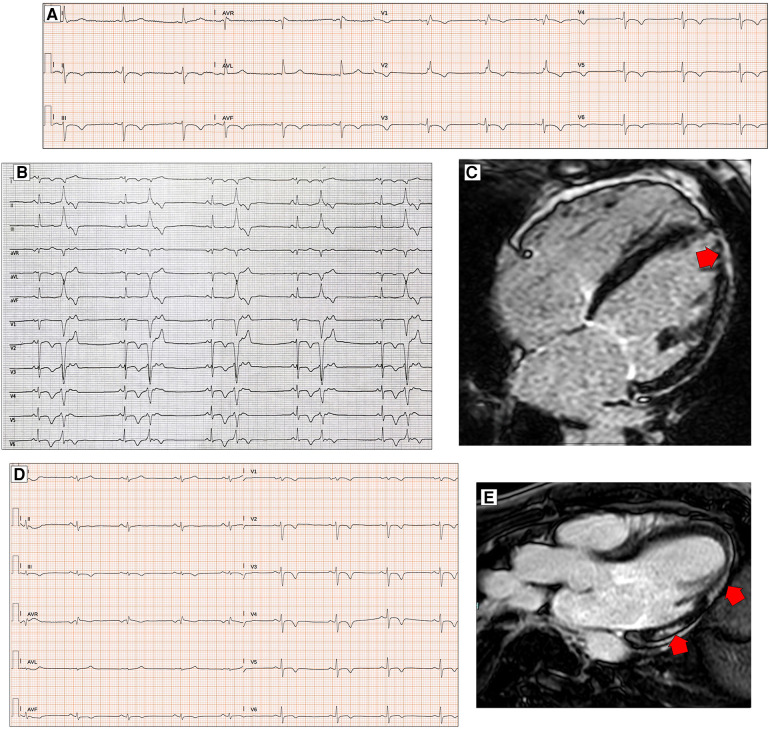
ECG characteristics in patients with arrhythmogenic cardiomyopathy. A 65-year-old PKP2 mutation carrier with arrhythmogenic right ventricular cardiomyopathy. ECG shows RBBB with T-wave inversion extending to the left precordial leads and inferior leads (**A**). ECG and CMR findings in a case of biventricular arrhythmogenic cardiomyopathy variant in a 34-year-old female. ECG showing LPFB, diffuse negative T waves, and monomorphic premature ventricular beats with LBBB/inferior axis morphology (**B**). Post-contrast CMR images in long-axis four-chamber view showing right ventricular dilation and subepicardial LGE involving the apical LV wall and the apical layer of the LV lateral wall (**red arrow, C**). ECG and CMR findings in a case of left dominant arrhythmogenic cardiomyopathy variant in a 35-year-old male with underlying PKP2 pathogenic variant. Basal ECG showing low QRS voltages in limb leads and T-wave inversion in both inferior and precordial leads (**D**). Post-contrast CMR images in long-axis three-chamber view showing subepicardial LGE involving the basal and mid-apical layers of the LV infero-lateral wall (**red arrows**, **E**). All the ECGs presented in the figure were performed at 25 mm/s with 1 mm/mV. ECG, electrocardiogram; CMR, cardiac magnetic resonance; LBBB, left bundle branch block; LGE, late gadolinium enhancement; LPFB, left posterior fascicular block; LV, left ventricular; RBBB, right bundle branch block.

The RBBB, complete or incomplete, is a frequent finding in ARVC. However, the morphology of RBBB is different from the classical form with a reduction in voltage of both r-waves, a reduction in the R/S ratio in V1–V2 when compared to RBBB in the absence of structural right heart disease, and also associated inverted waves with a greater extent than normal (above V3; Figure 4A) (64). The presence of a terminal activation delay (TAD) of the terminal portion of the QRS complex in right precordial leads is one of the more common ECG findings. Indeed, an increased duration of >55 ms from the nadir of the S wave to the end of the QRS when it returns to the isoelectric line is identified as an ECG diagnostic criterion in ARVD (65). In 2008, Cox et al. (66) demonstrated how the prevalence of activation terminal delay was significantly higher in baseline ECG of patients with ARVD/C than that in patients with idiopathic ventricular tachycardia from the right ventricular (RV) outflow tract. TAD has been associated with worse right ventricular function and greater right ventricular volume (67). It has to be noted that in the most recent “Padua Criteria,” the presence of the S wave upstroke delay in right precordial leads is classified as a minor criterion in the diagnostic workup of ARVC (55).

The epsilon wave is a reproducible, low-amplitude positive deflection at the end of the QRS complex, where the ST-segment begins. It manifests as a late depolarization of the free wall of the RV myocardium, particularly the epicardium and perivalvular region, and is mainly recorded in leads V1–V4 (68). In order to better identify the presence of epsilon waves on the electrocardiographic trace, Fontaine et al. identified the possibility of positioning the electrodes in a non-canonical manner [right arm (RA) over the manubrium, left arm (LA) over the xiphoid process, and left leg (LL) in the standard V4 position] to facilitate the visualization of the wave itself (69, 70). The epsilon wave may be isolated, but two or more waves may also be present at the QRS complexes of the same lead. However, due to its reduced prognostic impact, poor correlation with imaging criteria of pathology severity, low specificity, and marked inter-observer variability in its definition and identification, it has been downgraded from a major to a minor electrocardiographic criterion in the most up-to-date consensus on the topic (55).

The fQRS refers to the “slurs or notches” that appear on the R or S wave of the QRS complex, and it is an expression of a disarray of the electrical stimulus through the myocardium. It has been linked to myocardial scarring, free wall aneurysm and bulging, or the reduction of ventricular longitudinal strain at speckle-tracking evaluation (71).

Signal-averaged ECG (SAECG) is an amplified ECG deflection defined better by the Simpson method with XYZ electrodes, which can detect abnormal very late potentials (VLP) of low amplitude as an adjunctive expression of regions of slow conduction (68–72). These regions of electrical delay could be the substrate for ventricular arrhythmias due to a reentry circuit. They are found in almost 50% of ARVC patients and are even more frequent in severe forms of the pathology. However, due to the impractical electrocardiographic acquisition method to highlight SAECGs on the surface trace and their difficult definition, they have been abandoned as electrocardiographic criteria in more recent statements (55).

In almost 90% of ARVD patients, T-wave inversion in right precordial leads can be identified. The presence of T-wave inversion in right precordial leads (V1–V3) or beyond in patients with complete pubertal development (in the absence of complete RBBB) remains the only major electrocardiographic criterion in the Padua Criteria diagnostic definition of ARVD (55). As minor criteria, we find the presence of inverted T waves in leads V1 and V2 in individuals with completed pubertal development (in the absence of complete RBBB) and inverted T waves in V1–V4 in individuals with completed pubertal development in the presence of complete RBBB. Over the years, a correlation has been demonstrated between T-wave inversion and right ventricular dysfunction and dilatation evaluated on both echocardiogram and magnetic resonance imaging (54) (Figure 4B,C). Some studies have hypothesized the importance of the amplitude of inverted T waves in right precordial leads, particularly the prevalence of T waves of >2 mm in V1, found in approximately 90% of patients diagnosed with ARVC, as a diagnostic marker of the pathology itself. However, this electrocardiographic evaluation was not subsequently confirmed and did not find its way into the accepted diagnostic criteria of ARVC (73).

Finally, the importance of the presence of ventricular arrhythmias found incidentally on ECG or evidenced by prolonged electrocardiographic monitoring (Holter ECG) in the diagnosis of ARVC should be emphasized. In particular, a major diagnostic criterion is defined as the presence of frequent ventricular extrasystoles (500 per 24 h), non-sustained or sustained ventricular tachycardia of LBBB morphology. A minor criterion is the presence of frequent ventricular extrasystoles (500 per 24 h), non-sustained or sustained ventricular tachycardia of LBBB morphology with an inferior axis (“right ventricle outflow tract (RVOT) pattern”) (74, 75) (Figure 4B). Regarding this minor diagnostic criterion, in order to distinguish between ventricular arrhythmias caused by pathology and those typical of idiopathic ventricular arrhythmias originating from RVOT, Hoffmayer et al. (76) pointed out in 2011 that certain features, such as the presence of notches on ventricular extrasystole complexes in at least one of the precordial leads, a QRS duration in D I of >120 ms, a late transition (from V5), and the earliest-onset QRS in lead V1, were all predictors of ventricular arrhythmic events related to ARVC when compared with subjects with idiopathic ventricular arrhythmias originating from the right infundibulum. As pointed out already in the early 2000s by Fontaine et al., a correct identification of the origin of ventricular arrhythmias, the clinical and prognostic impact of the arrhythmias themselves, and their possible ablation treatment are also crucial (77).

### 3.2. ECG abnormalities and ventricular arrhythmias in ALVC

The electrocardiographic diagnostic criteria for ALVC are less well-defined, probably due to the more recent identification of this pathology. There are no major criteria, but all the electrocardiographic parameters are classified as minor criteria. Low QRS voltages (LQRSV) (<0.5 mV peak to peak) in limb leads in the absence of obesity, emphysema, or pericardial effusion may be the most sensitive sign of LV involvement in ACM (78, 79) (Figure 4D,E). There was a statistically positive correlation between low QRS voltages in limb leads and the amount of left ventricular late gadolinium enhancement (*P* < 0.001) (63). This type of electrocardiographic pattern occurs more frequently in patients who carry certain mutations, such as those in the phospholamban and filamin C genes, which are typically associated with left ventricular involvement in the context of purely left or biventricular ACM (26, 80, 81). The reduction of voltages in limb leads is a parameter with low sensitivity because it often indicates an advanced pathology in which fibro-adipose replacement is already relatively extensive compared with the overall ventricular mass. On the other hand, the reason why the electrocardiographic manifestation is confined almost exclusively to the limb leads is unclear.

The basal infero-posterior region, supplied by the posterior fascicle, is the ventricular myocardial portion often early affected in several cardiomyopathies, including ALVC, as evidenced by the presence of LGE on MRI studies (82). Based on this hypothesis, Calò et al. (18) conducted a retrospective study analyzing ECGs of young patients who had died of SCD or had suffered aborted cardiac arrest (ACA) and compared them with apparently healthy patients, focusing their attention on the hypothesis that LPFB could be an early electrocardiographic marker of structural pathology, particularly related to the future development of ALVC (Figure 4B,C). The prevalence of LPFB in patients with resuscitated SCD was 100-fold higher than in the control group, and in patients with ACA, CMR analysis showed structural pathology in all the subjects (mainly LGE in the infero-lateral and infero-septal location). These data need to be confirmed in further studies to allow us to include this parameter in the diagnostic criteria of ALVC (83).

Another minor electrocardiographic criterion for the diagnosis of ALVC is the presence of inverted T waves in the left precordial leads (V4–V6). The specificity of this finding is not high because the presence of deep negative T waves in these leads, especially when associated with inversion of the same waves in the right precordial leads, could be an expression of arrhythmogenic pathology with a predominantly left component (Figure 4B–E). It could instead be the expression of a biventricular pathology or even the electrocardiographic manifestation of severe right ventricular dilatation with alterations of ventricular geometry, resulting in a pathologic electrocardiograph tracing extended also to the non-specifically right leads, as demonstrated by several correlation studies between ECG and imaging methods (84, 85).

The last minor criterion for left ventricular involvement in the context of arrhythmogenic heart disease is the presence of frequent ventricular extrasystoles (500 per 24 h), non-sustained or sustained ventricular tachycardia with an RBBB morphology (excluding the “fascicular pattern”) (55).

Evidence from a recent study focused on ventricular arrhythmias in athletes has highlighted specific characteristics that allow for the differentiation of extrasystoles in structurally healthy hearts from extrasystoles in subjects with left ventricular scar (arrhythmogenic cardiomyopathy) (86).

## 4. Hypertrophic cardiomyopathy

Although 5%–10% of patients with hypertophic cardiomyopathy (HCM) present an electrocardiographic trace within normal limits, the ECG plays a key role in raising the first diagnostic suspicion of hypertrophic cardiomyopathy (87). There are several electrocardiographic manifestations that may suggest imaging tests to confirm the diagnosis (88, 89).

**Figure 5:**
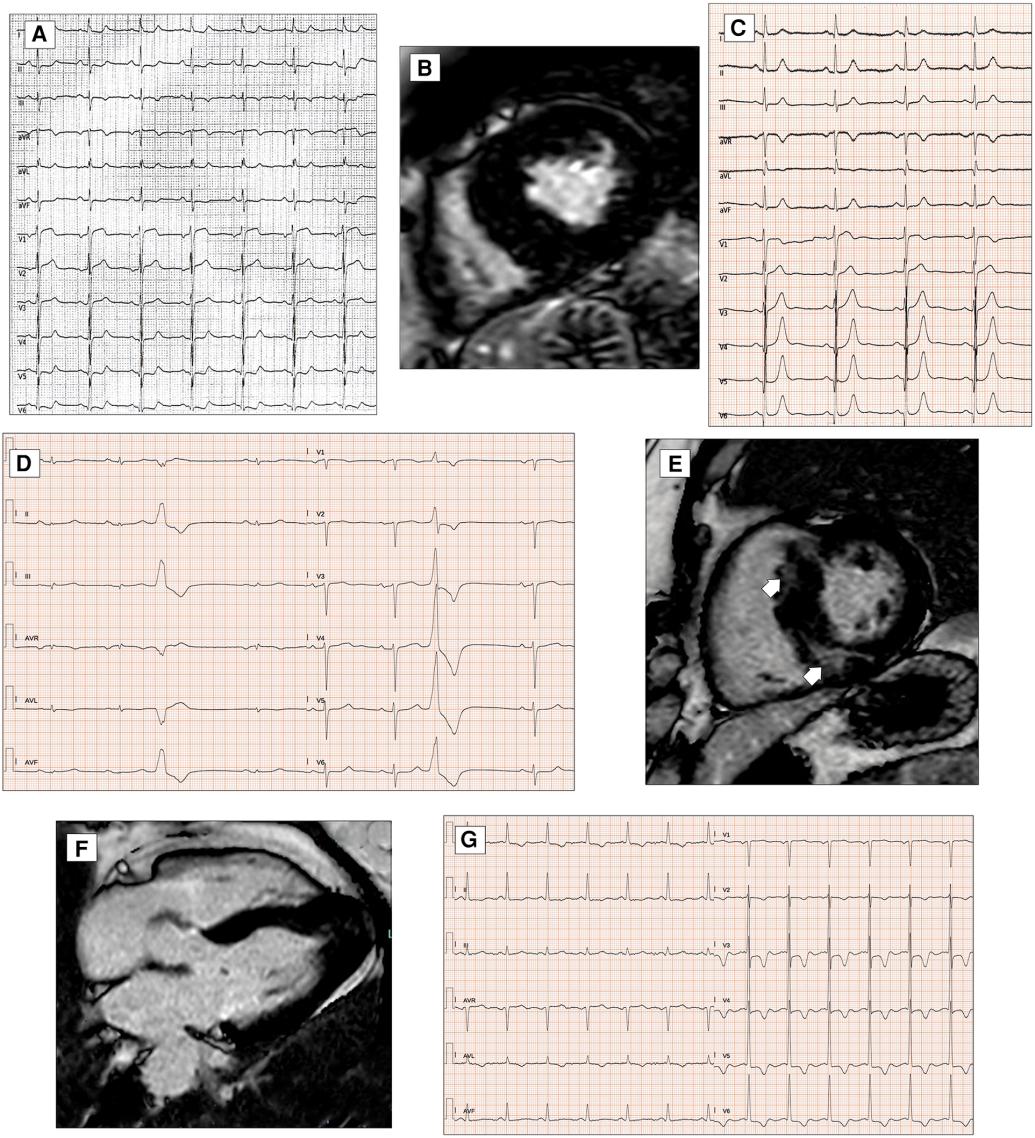
ECG abnormalities in patients with hypertrophic cardiomyopathy. ECG and CMR findings in a 78-year-old patient with obstructive HCM. Twelve-lead ECG showing ST-segment alterations in infero-lateral leads (**A**). Post-contrast CMR images in short axis showing hypertrophic septal asymmetric cardiomyopathy (**B**). Pathological Q waves in lateral leads and signs of LVH (Sokolow–Lyon criteria) in a 55-year-old male with non-obstructive HCM (**C**). ECG and CMR findings in a 56-year-old male with non-obstructive HCM. Note the low QRS voltages in limb leads at 12-lead ECG (**D**). LGE is found at the insertion points of the interventricular septum with hazy mid-wall enhancement in areas of hypertrophy (**white arrows, E**). CMR and ECG findings in a case of apical HCM in a 56-year-old female. CMR reveals obliteration of the cavity at the apex and the apical displacement of papillary muscles (**F**). ECG shows signs of LVH and deeply inverted T waves in precordial leads and lateral leads (**G**). All the ECGs presented in the figure were performed at 25 mm/s with 1 mm/mV. ECG, electrocardiogram; CMR, cardiac magnetic resonance; HCM, hypertrophic Cardiomyopathy; LGE, late gadolinium enhancement; LVH, left ventricular hypertrophy.

The baseline ECG may show alterations in the P-wave, with the possibility of signs of atrial hypertrophy, and alterations in the QRS complex and ST-T tract (Figure 5A,B). During prolonged ECG monitoring, the presence of atrial fibrillation and isolated or repetitive ventricular extrasystoles is common (90).

It should be emphasized that ECG abnormalities in HCM patients may precede echocardiographic findings of the disease (79, 81). This means that the subject may carry the mutation responsible for the disease, but the phenotypic expression of the pathology has not yet manifested clinically (81).

Left atrial enlargement expressed by a bifid and large P-wave (total duration of >110 ms) may reflect the presence of elevated diastolic filling pressure and could be a negative predictor for the future development of atrial fibrillation. Signs of right atrial enlargement, such as a P-wave amplitude of >2.5 mm in inferior leads and >1.5 mm in V1–V2, are less frequent and often are the expression of an inveterate presence of high pulmonary capillary pressures in more advanced disease (91).

The QRS complex in patients with HCM is often abnormal. The classic signs of left ventricular hypertrophy, such as the Sokolow–Lyon and Cornell signs (SV3 + RaVL with a cutoff for LHV of >2.0 mV in women and >2.8 mV in men), are present in isolation in almost 2% of the patients (Figure 5C), contrary to what one might think (92). A small observational study showed that all ECG voltage criteria had a poor correlation with left ventricular mass and maximal thickness measured by MRI (93). Conversely, in patients with HCM, high-voltage QRS complexes are often accompanied by other pathological morphologies of the QRS complex and the ST-T tract.

A recent study published by Pelliccia et al. (94) demonstrated that low QRS voltages are present in 11% of patients with HCM and are associated with a larger extent of LGE on MRI (Figure 5D,E); furthermore, after a follow-up of 4.5 ± 2.6 years, the presence of LQRSV in HCM are associated with a higher incidence of functional deterioration, stroke, and ICD implant.

The presence of an fQRS complex has been correlated with the presence of myocardial fibrosis detectable on MRI (95). In 2015, a study by Konno et al. (96) demonstrated how the number of leads that showed the presence of a fQRS was the best predictor of the evidence of LGE on MRI.

Pathological Q waves are present in almost 40% of patients with HCM (Figure 5C). This could be another electrocardiographic expression of the presence of myocardial fibrosis or, as some have suggested, could also be due to abnormal activation of the septal or free wall portion at the level of the base of the left ventricle (83). The evidence of LGE on MRI was alternatively correlated with the ECG finding of Q waves in patients with HCM. The presence of LGE on MRI and evidence of Q waves on ECG have shown varying degrees of correlation over the years (97). In 2007, Papavassiliu et al. (98) hypothesized that not so much the presence of LGE but the distribution and, in particular, the segmentarity, the transmurality, and also the ratio of the thickness of the interventricular septum and posterior wall assessed on MRI were the real determinants of the presence or absence of Q waves on ECG. Chen et al. (99) demonstrated that the presence of pathological Q waves in lead D III, in the context of other signs of left ventricular hypertrophy, may distinguish patients with HCM from athletes, regardless of other ECG markers.

Not particularly useful for diagnostic purposes due to low sensitivity and specificity, predominantly left intraventricular conduction delays are considerably more frequent in the postoperative phase after myectomy or alcohol ablation of the septum, used for resolving the gradient at the ventricular outflow in obstructive forms and advanced disease (83). On the other hand, repolarization abnormalities are very common, primarily ST strain, defined as ≥1 mm concave downsloping ST-segment depression with asymmetrical T-wave inversion in the lateral leads, ST-segment elevations in antero-lateral leads known as the “pseudo-STEMI” pattern, and deep T-wave inversion (≥2 mm) in infero-lateral leads with, in some cases, the so-called “giant T waves” (>10 mm), a typical finding in the apical form of HCM (89, 100) (Figure 5F,G). In 2013, the analysis of Delcrè et al. (83) demonstrated that abnormalities of repolarization were seen in almost 50% of patients with HCM. The same study demonstrated how the number and severity of depolarization and repolarization abnormalities were directly related to the CMR findings. Recently, it has been shown that the presence of J waves, defined as the presence of J-point elevation with an end-QRS notch or slur (the J wave) on the downslope of a prominent R-wave of ≥1 mm involving ≥2 leads (commonly in infero-lateral leads), excluding V1–V3, was positively associated with cardiac events such as sudden cardiac death, ventricular arrhythmias, and ventricular fibrillation (101).

The proarrhythmic risk of patients with HCM is also increased due to QT interval prolongation, which occurs in about one-eighth of patients with HCM (102). The cause of the prolongation of the QT interval beyond 480 ms in these patients can be attributed to alterations in the ion channels that interact with the cellular proteins forming the myocardial sarcomeric structure or to fibrous substitution and disarray phenomena that may result in action potential dispersion (103).

In the stratification of arrhythmic risk and the risk of evolution to end-stage forms, evaluation of the evolution of the electrocardiogram trace is also important in patients with HCM. In fact, it has been shown that there is often an evolution of the electrocardiogram tracing that goes hand in hand with the structural evolution of the myocardium due to fibrotic replacement of the myocardium for phenomena of necrosis and apoptosis (104). For example, Pennacchini et al. (105) showed how the aneurysmal evolution of hypertrophic pathology at the apical level tended to develop peculiar electrocardiogram patterns such as persistent ST-segment elevation, constant J-point elevation, reduced T-wave amplitude, and also reduced amplitude of QRS complex voltages. Also, in the French REMY registry (106), some parameters such as voltage reduction, presence of Q waves, QT interval prolongation, and presence of ventricular extrasystoles with RBBB morphology were predictors at multivariate analysis of worse outcome, although at the adjusted endpoint statistical analysis, they did not reach statistical significance. As pointed out by Olivotto et al. (82), HCM can have different stages within the patient's clinical history, with a phenotype that is initially concealed and later manifested, and in even more advanced stages can have a potentially negative evolution from both a structural and electrocardiographic point of view. The electrocardiogram thus becomes a valid and early tool for assessing the different stages in both diagnostic and prognostic terms.

## 5. Restrictive cardiomyopathy

Under the definition of restrictive heart disease falls a very various spectrum of pathology that has its common denominator in certain features such as advanced diastolic dysfunction, reduced ventricular chamber size, and dilation of the atrial chambers in the presence or absence of increased parietal thicknesses. It should be emphasized that a restrictive pattern does not always identify a restrictive cardiomyopathy but may be the expression of a worsening evolution of a dilated or hypertrophic cardiomyopathy or vice versa it may be an early manifestation of pathology that could potentially evolve into a hypokinetic or dilatative phenotype (101). Furthermore, in the broad spectrum of restrictive cardiomyopathies, we often find a different pathogenesis and phenotypic expression. In particular, we find endomyocardial fibrosis (e.g., in forms of hypereosinophilic syndrome or endomyocardial fibroelastosis) typically with a non-hypertrophic phenotype, genetic forms (sarcomeric and non-sarcomeric), or secondary to radiotherapy systemic sclerosis typically presenting interstitial fibrosis and lastly infiltrative forms, the most frequent of which are amyloidosis or storage disorders (e.g., glycogen, Anderson–Fabry, Gaucher disease, Danon disease, hemochromatosis) (107). This group of pathologies, mainly hemochromatosis and cardiac amyloidosis (CA) that typically manifest with a hypertrophic phenotype on imaging examinations, often does not disclose electrocardiographic signs consistent with increased parietal thickness. Precisely in view of the presence of hypertrophy not due to actual myocardiocyte growth but to fibrous replacement, increased extracellular matrix, or intracellular swelling with material characterized by reduced electrical excitability compared with myocardial cells, the electrocardiographic manifestation is more often the presence of low QRS voltages rather than typical signs of ventricular hypertrophy (24, 106, 107). Precisely because of the heterogeneity of the pictures associated with restrictive forms of cardiomyopathy, it is not possible to identify a common electrocardiographic characteristic. However, some of the conditions already mentioned have electrocardiographic peculiarities. For instance, in Danon's disease, there are some characteristic electrocardiographic patterns as extremely high voltages in the different leads as well as presence of pre-excitations with the frequent finding of multiple atrial accessory pathways or ventricular fascicles on electrophysiological study (108).

### 5.1. ECG findings in Fabry disease

Anderson–Fabry disease (AFD) is a genetic lysosomal storage disorder characterized by progressive intracellular accumulation of glycosphingolipids, resulting in multi-organ involvement. Despite the wide range of clinical phenotypes observed in AFD patients, the ECG plays an important role in detecting cardiac involvement.

In childhood and adolescence, subtle ECG changes can be detectable, as LV mass at the upper limits of the normal range is reported for the general population, with males more frequently affected than females (109). Moreover, a short PQ interval and repolarization abnormalities (110, 111) may precede LV hypertrophy, which generally manifests after the third or fourth decade of life.

**Figure 6:**
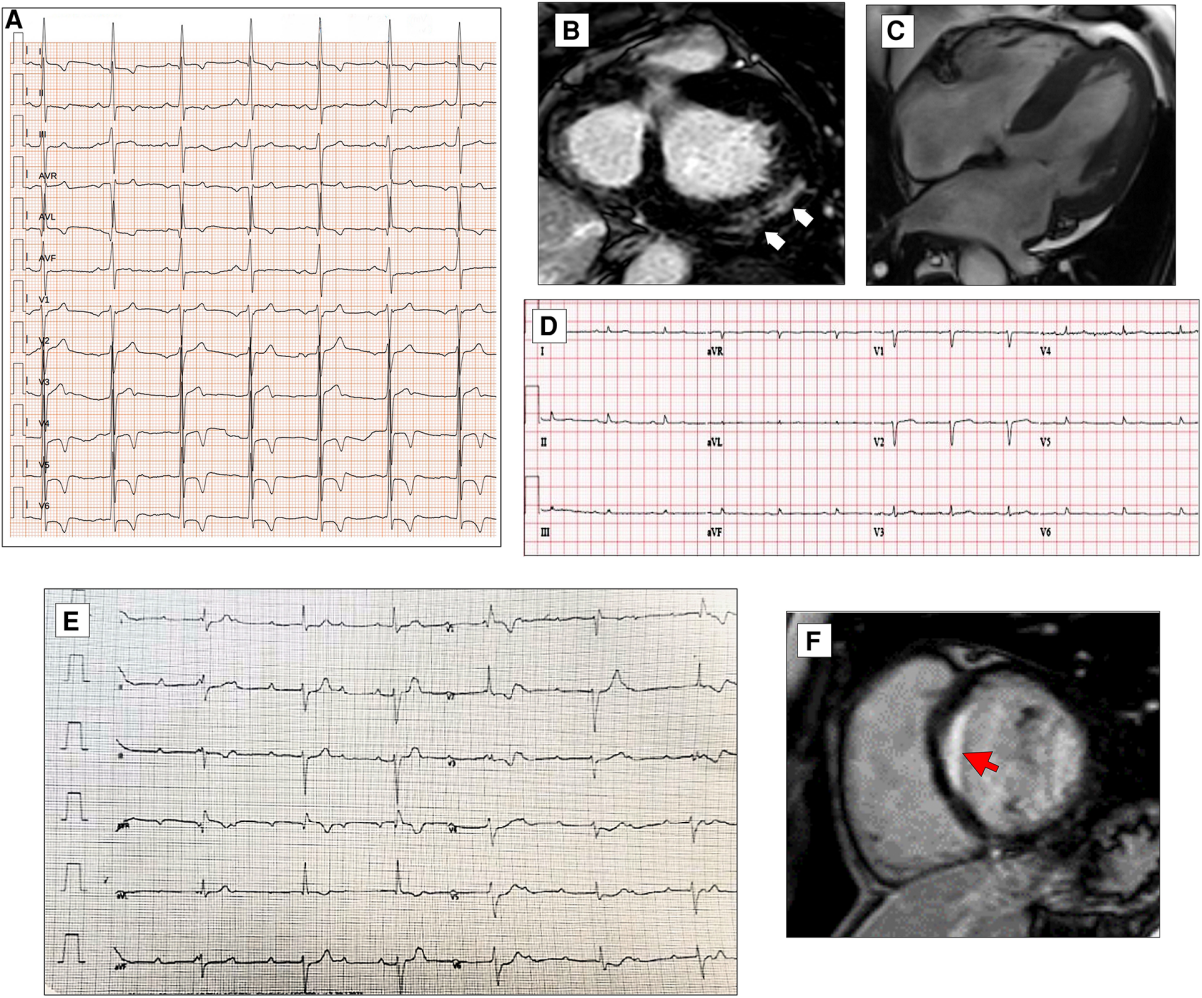
ECG characteristics in HCM phenocopies and sarcoidosis. ECG and CMR findings in a 45-year-old patient with Anderson–Fabry disease. Twelve-lead ECG displaying signs of LVH and deep T-wave inversion in D1-aVL and left precordial leads (**A**). Post-contrast CMR images in short axis showing LVH and LGE within the basal postero-lateral LV wall (**white arrows**, **B**). CMR and ECG features of a patient affected by cardiac ATTR amyloidosis. End-diastolic frame of cine CMR sequence in long-axis four-chamber view showing moderate LVH (**C**). Basal ECG showing low QRS voltages both in limb and precordial leads (**D**). ECG of a 30-year-old female with cardiac sarcoidosis. Note the atrioventricular dissociation with wide QRS escape rhythm (**RBBB and LAFB morphology, E**). CMR of the patient in panel E reveals transmural LGE in the basal septum (**red arrow**, **F**). All the ECGs presented in the figure were performed at 25 mm/s with 1 mm/mV. ECG, electrocardiogram; CMR, cardiac magnetic resonance; HCM, hypertrophic cardiomyopathy; LAFB, left anterior fascicular block; LGE, late gadolinium enhancement; LVH, left ventricular hypertrophy; RBBB, right bundle branch block.

In adults, ECG signs of LV hypertrophy (high-voltage criteria, left ventricular strain pattern, and T-wave inversion in the precordial leads; Figure 6A,B) are the most common features of cardiac disease, reflecting the progressive development of overt structural heart abnormalities. In the majority of cases, the manifestation of LVH is accompanied by other ECG abnormalities, such as a short PR interval, which is most probably due to accelerated intra-atrial conduction (112–114). With disease progression, the extent of conduction system infiltration increases, causing PQ interval prolongation, sinus, and atrioventricular node dysfunction (115).

Older patients show prolonged QRS complex and intraventricular conduction delay, resulting from the increased time of ventricular depolarization (116). In addition, ST-segment depression and T-wave inversion in infero-lateral leads may be observed, reflecting the presence of myocardial fibrosis and increased risk for arrhythmic complications.

Regular ECG evaluation and prolonged ECG monitoring are strongly recommended in AFD patients in order to identify high-risk patients who can benefit from antiarrhythmic drugs or PMK/ICD implantation (117).

### 5.2. ECG findings in amyloidosis

The most striking electrocardiographic abnormality in patients with CA is the reduction of QRS voltages, particularly in the limb leads, and the disproportion between low QRS voltages and the increased LV thickness at echocardiography (118).

The ECG can raise or support the clinical suspicion of CA by revealing various red flags, such as atrioventricular conduction disturbances, pseudoinfarction patterns, and LQRSV, defined as a QRS amplitude of ≤5 mm (0.5 mV) in all peripheral leads, including both negative and positive components (119–121) (Figure 6C,D).

Low QRS voltages can be observed in up to 60% of patients with CA, more frequently in amyloid light-chain cardiac amyloidosis (AL-CA) than in transthyretin cardiac amyloidosis (ATTR-CA), and may hypothetically reflect the burden of amyloid infiltration in the heart (122).

Beyond their diagnostic value, LQRSV also have a prognostic role in clinical practice and have been demonstrated to be associated with increased mortality (63%) in individuals free of any cardiovascular disease (123). LQRSV reflect an advanced disease stage and independently predict cardiovascular death (124). The prognostic value of LQRSV was also confirmed by Kristen et al. (125), who found that LQRSV were independently associated with decreased survival in a combined cohort of patients with AL-CA and ATTR-CA.

In terms of conduction defects, patients with ATTR-CA also present with a higher prevalence of conduction defects, including first-degree AV block or higher and intraventricular delay.

Over one-third of wild-type ATTR-CA patients experience AF compared with 20% of the ATTR-CA variant and the only 6% of those with AL-CA. AF with slow ventricular response, AV block, and intraventricular delay were also more common among ATTR-CA patients, leading to a significant prevalence of device implantation before diagnosis. Conversely, AL-CA subjects more often presented in sinus rhythm and displayed the typical low-voltage pattern at diagnosis (126).

A plausible interpretation is that ATTR-CA behaves as a progressive cardiomyopathy characterized by slow amyloid deposition within the atria, the ventricles, and the conduction system, while AL-CA manifests as an acute myocarditis with early symptom onset and rapid disease progression to end-stage heart failure, despite lesser degrees of infiltration, due to the toxic effects of AL chains.

In a large cohort of AL-CA and ATTR-CA patients, 8.9% received a PMK within 3 years after diagnosis. A history of atrial fibrillation, PR of >200 ms, and QRS of >120 ms predicted future PMK implantation (127).

## 6. ECG in acquired forms of cardiomyopathy: sarcoidosis

ECG abnormalities are common in patients with clinically manifest cardiac sarcoidosis (CS) (128). These abnormalities often occur prior to the development of cardiac events and are associated with subsequent development of severe cardiac manifestations such as complete atrioventricular block (AVB), ventricular tachyarrhythmias, and heart failure. Further evaluation using advanced imaging modalities such as MRI and positron emission tomography should be considered in patients with extracardiac sarcoidosis who have electrocardiographic abnormalities (129, 130).

AVB is one of the most common cardiac manifestations resulting from infiltration of the intraventricular septum due to sarcoid granuloma or, at a later stage, scar tissue (Figure 6E,F). Complete atrioventricular block occurs in 20%–30% of cardiac sarcoidosis cases, and a prolonged PR interval was associated with the onset of cardiac manifestations (120). According to the 2014 Heart Rhythm Society consensus statement (131), unexplained Mobitz II or third-degree atrioventricular block in young patients aged less than 60 years should raise suspicion for cardiac sarcoidosis.

The RBBB is another common ECG feature, included in the minor criteria for the diagnosis of sarcoidosis, and is associated with the development of cardiac events (120). The LBBB is rare and has been associated with the presence of cardiac dysfunction (120).

Fragmented QRS is frequently found in patients with CS and in 8% of patients with extracardiac sarcoidosis (132). It is associated with ventricular tachyarrhythmias and the occurrence of cardiac events in extracardiac sarcoidosis (120). A relationship between the leads where fragmented QRS is present and the sites of LGE in MRI in cardiac sarcoidosis is reported (133).

Another ECG feature reported in CS patients is the epsilon wave of the QRS complex, which makes differentiation from ARVC. This epsilon wave is described as more prominent. A recent algorithm including PR prolongation and the surface area of the maximum R′ wave in leads V1 through V3 of >1.65 mm^2^ is proposed to distinguish CS from ARVC. This QRS terminal activation in precordial leads V1 through V3 may reflect disease-specific scar patterns (134).

The frequency of ST-segment abnormalities is high in patients who develop cardiac events (36%). These abnormalities may reflect damaged myocardium and were present prior to the development of heart failure in all patients (120).

Other repolarization abnormalities reported in some studies include increased QT dispersion (135), the presence of microvolt T-wave alternans (136), higher T-wave amplitude in lead aVR (137), T-wave inversion (128), and a longer interval of T-peak to T-end (126).

## 7. Conclusion

Over the past 50 years, knowledge about cardiomyopathies has exponentially increased due to the introduction of new imaging techniques, genetic analysis, specific laboratory tests, and endomyocardial biopsy, which have changed the clinical approach to these diseases.

The use of imaging techniques such as echocardiography, CMR, and nuclear imaging has allowed us to define cardiac morphology and function. In addition, thanks to tissue characterization, it is possible to obtain a differential diagnosis and recognize specific forms of myocardial disease (138).

Genetic testing offers the possibility to identify specific forms of cardiomyopathies due to single-gene mutations and to discover the great variability in expression of these genetic forms that can show different phenotypes, even in the same family (139).

These tools are useful and required for an accurate diagnosis, but they are not always available in clinical practice and are accessible only to reference centers. The electrocardiogram is a simple, reproducible, widely accessible, and low-cost technology useful to the physician for the first-line screening of symptomatic and asymptomatic patients. It can provide important diagnostic information to identify patients worthy of further investigation.

Distinguishing between ECG abnormalities that may suggest underlying pathology represents a basic knowledge target for the clinical cardiologist. In this review, we showed the ECG abnormalities typical of each cardiomyopathy. In addition, we highlighted how some of these ECG findings, in association with other specific clinical features, can suggest a specific diagnosis and guide subsequent diagnostic steps and management.

We also showed how the role of ECG in sudden death risk stratification is central. Different cardiomyopathies have their own risk score that includes morpho-functional features, genetic mutations, and ECG abnormalities. However, the clinical utility of these scores is still uncertain and will need to be further investigated.
